# Supplementary material for: Complexome profiling of the Chlamydomonas psb28 mutant reveals TEF5 as an early PSII assembly factor
Source: Plant Cell. 2025 Mar 17;37(6):koaf055. doi: 10.1093/plcell/koaf055 (PMC12164590; doi:10.1093/plcell/koaf055)
Supplement: koaf055_Supplementary_Data [file koaf055_supplementary_data.zip › psb28_interactive.zip!/plots/intensity/qualityPlots/peptides/TEF30_Cre01.g031100.t1.2.html]

### Cre01.g031100.t1.2

Trivial name: TEF30  
SeqCoverage [median]: 38.60  
Mass: 37.03 kDa  
  
 

| Sequence | MissedCleavage | Mass [Da] | wtIntensity | muIntensity | Score |
| --- | --- | --- | --- | --- | --- |
| AENFGQIMYAIR | 0 | 1411.69 | 2463.48 | 2226.86 | 108.57 |
| AGGNYGAGTK | 0 | 894.42 | 10454.41 | 1080.76 | 183.43 |
| AGGNYGAGTKEIQAR | 1 | 1491.74 | 3654.42 | 1011.03 | 102.50 |
| AGNIDAR | 0 | 715.36 | 15435.10 | 2479.84 | 127.84 |
| ATFGAFGNMFNK | 0 | 1303.60 | 6862.68 | 2988.20 | 116.05 |
| ATFGAFGNMFNKEK | 1 | 1560.74 | 2699.53 | 0.00 | 50.56 |
| EMFDDALAK | 0 | 1038.47 | 3790.63 | 1710.16 | 117.40 |
| GNDGGAYIIEVNPK | 0 | 1445.72 | 16200.16 | 7252.88 | 160.38 |
| NFVGDNFSR | 0 | 1054.48 | 18426.29 | 4122.71 | 149.57 |
| NTPIYK | 0 | 734.40 | 1163.85 | 0.00 | 90.99 |
| NYGDLSALEEEGLDAAEK | 0 | 1922.87 | 1972.52 | 0.00 | 193.06 |
| SGTVYMK | 0 | 784.38 | 2197.88 | 0.00 | 107.43 |
| SLDAAMLSGFDNYDQIR | 0 | 1914.88 | 0.00 | 841.04 | 105.17 |
| SLDAAMLSGFDNYDQIRR | 1 | 2070.98 | 8426.87 | 2765.55 | 77.75 |
|  |  |  | 93747.8 | 26479.0 |  |
